# Extra-Uterine Pregnancy

**Published:** 1878-02

**Authors:** M. H. Van Ripen

**Affiliations:** Kankakee, Ill.


### NOTES FROM PRIVATE PRACTICE.

*Extra-Uterine Pregnancy.*

Mrs. R——, aged 26, a spare woman of medium height, married nine years; has never been previously pregnant; since the first establishment of her menses, she has occasionally had severe attacks of flooding, except during the last two years, since which time she has been quite regular, except for the interruption caused by pregnancy.

My attention was first called to her about the middle of September, 1876. She was then the subject of severe and persistent nausea and vomiting, and I was told her menses had failed to make their appearance at her last period. I diagnosticated pregnancy, and gave her oxalate of cerium and subnitrate of bismuth, by which these symptoms were relieved. She was left, however, with distressing indigestion; her food passed through the alimentary canal almost without change, and she became very much emaciated. My prescriptions for this were pepsin, and warm water baths followed by friction. These mitigated the symptoms to some extent, but she still had loathing of food, and almost every aliment distressed her. For constipation, she took carbonate of magnesia and clysters of soap, salt and water.

In January, 1877, she commenced complaining of pains in the right side, just under the floating ribs. This continued, with more or less severity until the 10th of August following. December 25th, she was taken with flooding, which continued to recur at intervals in spite of gallic acid, acetate of lead and refrigerants. About the 1st of March, the breasts began to enlarge, and by the middle of the month they were filled with milk to such a degree that it flowed from the nipples profusely enough to wet her clothing.

The abdomen was not enlarged, but, owing to the great emaciation, was sensibly diminished in size, except in the region of the pains described above. In that locality there was a distinct prominence, or tumor, which was very sensitive to pressure. About the 1st of May, the patient was seized with an uncontrollable diarrhœa, which lasted, with varying severity, for two months. On the 26th of May, after great pain, a quantity of very offensive pus was discharged from the bowels. This continued to pass with every evacuation until the *debris* of the fœtus came away. Pains like labor pains commenced on the 9th of June, and lasted until the 25th of that month. The pains at that time increased in severity, one or two occurring every day. July 20th, a pain of very great severity took place, and lasted as much as thirty minutes, and caused the patient to faint. From that time there was constant pain until August 1st, when a fœtal rib was passed from the rectum. On the 2d of August, two ribs and a femur were expelled ; and on the 4th of that month most of the trunk. On the 10th, the skull bones, three ribs and a femur were expelled. From that time all pain ceased, and the patient became comfortable. Her appetite returned, her strength improved, she gained flesh—in fact convalescence from her protracted suffering was at once inaugurated. Her diet at this time consisted of beef tea, chicken broth, farina, rice, milk, broth and meat of wild birds, with brown bread. She took clysters of carbolic acid and glycerine. She is now taking the elix. ferri et calcis phosphatis with strychnia and fluid extract of calisaya.

The following is the measurement of some of the expelled bones : The parietal bone measures two inches in length by one and three-eighths in breadth ; the frontal is one and three-quarters inches by one and three-eighths ; the occipital, one inch by one and a half ; the femur is one and three-quarters inches long without the apophyses, which are gone. I think the perforation of the intestines occurred in the ascending colon.

Respectfully,

M. H. Van Ripen, M. D.

Kankakee, Ill., Dec. 1, 1877.

